# Editorial: Technologies in smallholder poultry development: characterization, utilization, conservation, and improvement

**DOI:** 10.3389/fgene.2023.1288181

**Published:** 2023-09-13

**Authors:** Abdulmojeed Yakubu, Oladeji Bamidele, Aranganoor Kannan Thiruvenkadan, Moses Okpeku, Senol Çelik

**Affiliations:** ^1^ Department of Animal Science, Faculty of Agriculture, Centre for Sustainable Agriculture and Rural Development, Nasarawa State University, Keffi, Nigeria; ^2^ International Livestock Research Institute (ILRI), Ibadan, Nigeria; ^3^ Department of Biological Sciences, Kings University, Odeomu, Nigeria; ^4^ Department of Animal Genetics and Breeding, Veterinary College and Research Institute, Salem, Tamil Nadu, India; ^5^ Discipline of Genetics, School of Life Sciences, University of Kwa-Zulu Natal, Durban, South Africa; ^6^ Department of Animal Science, Faculty of Agriculture, Bingöl University, Bingöl, Türkiye

**Keywords:** smallholder-farming sector, poultry, genetics, innovative approaches and methods, improved production, food security

Smallholder poultry production accounts for about 80% of the poultry flock in low- and middle-income countries. The birds play important roles in rural livelihoods, food security, household income, culinary and traditional medicine and strengthening of social relationships (Li et al., 2020; Bamidele and Amole, 2021; OchoraKasima et al., 2023). Despite the benefits derived from Smallholder poultry production systems (SPPS), the production as well as the productivity is low. The low performance of the birds could be attributed mainly to low genetic potential, genetic erosion occasioned by indiscriminate crossbreeding, poor nutrition, health and housing and vagaries of climatic factors (Samaraweera et al., 2021; Birhanu et al., 2021; Wilson et al., 2022). This, according to Birhanu et al. (2023), necessitates poultry research and development using innovative and technological approaches in genetics, feed supply, health services, housing, and public-private partnership to deliver integrated innovation packages. The outcome will not only boost production, but ensure increased productivity and profitability of the SPSS.

This Research Topic (*Technologies in smallholder poultry development: characterization, utilization, conservation, and improvement*) centred on phenotypic and molecular characterization, quantitative and population genetics, genetic/genomic/proteomic evaluation and application of classical phenotyping methods to health, nutrition, production and reproduction including the interaction between the environment and poultry species. The ten articles contributed to this Research Topic are summarized below:

Feather pecking, which is a complex trait, and partly under genetic control, remains an important welfare and economic Research Topic in chickens. This prompted the work of Mott et al. to identify a potential key regulator for this behavioural disorder in laying hens. The study found that the increased propensity of laying hens to perform feather pecking could be due to lack of CD4 T cells and gamma-Aminobutyric acid (GABA) receptors. Therefore, the authors propose KLF14 as a clear candidate regulator for the expression of genes associated with the pathogenic development.

Housekeeping genes (HKGs) usually are used as the reference (internal controls) to evaluate and compare abundances of mRNA expression of target genes in different cells or tissues of animals. Due to the dearth of information on chicken HKGs, Hasanpur et al. tested most of the reliably expressed genes (REGs) for stability in 16 important chicken tissues (skin, adipose, blood, brain, bursa of Fabricius, heart, liver, lung, kidney, muscle, duodenum, ileum, jejunum, ovary, spleen, and trachea) using at least three RNA-seq datasets per tissue. The authors discovered a total of 6, 13, 14, 23, and 32 validated housekeeping genes (V-HKGs) as the most stable and suitable reference genes for muscle, spleen, liver, heart, and kidney tissues, respectively. These V-HKGs could be exploited in more accurate normalization for future expression analysis of chicken genes.

The Tagray region in Ethiopia is an ancient entry route for domestic chickens’ entry into Africa. The oldest African chicken bones were found in this region, dating back to around 800-400BCE. The region has a high chicken-to-human population ratio and diverse geography. Following the introduction of exotic chickens, the proportion of indigenous chickens in the region has decreased to 70%. The study by Gebru et al. used Ecological Niche Modelling to characterize the habitats of 16 indigenous village chicken populations in Tigray, identifying four main chicken agro-ecologies and potential indigenous Tigrayan chicken ecotypes. This information can guide conservation and breeding improvement initiatives for indigenous Tigrayan chickens.

The study by Kanakachari et al. investigated the difference in muscle development, egg production, and plumage colors between native and broiler chickens. The researchers conducted a microarray analysis using the 7th-day embryo and 18th-day thigh muscle of improved Assel broiler chickens, respectively. They selected 24 candidate reference genes and isolated total RNA from the chickens to study their expression profiles using real-time quantitative PCR. The study identified differentially expressed genes that regulate muscle growth, myostatin signaling and development fatty acid metabolism, and other pathways in improved Assel chickens. The findings may be used to improve muscle development, differentiation, egg production, protein synthesis, and plumage formation in native chickens and optimize growth in broiler chicken.

Improved tropically adapted birds have been reported to be suitable for backyard poultry production. In the light of this, Bamidele et al. introduced improved, dual-purpose chicken genetics into the smallholder farming households (SFH) as a helpful intervention to mitigate the impact of COVID-19 pandemic. The study was conducted in three states in Nigeria, each representing a distinct agroecological zone. The birds, which were managed under semi-scavenging production system, were evaluated for growth, survivability and profitability. The body weight of Noiler and FUNAAB Alpha chickens appeared similar. Agroecology and genetics significantly affected growth and survivability of the birds. Profitability was higher in Nasarawa state, followed by Kebbi and Imo. The study, therefore, posits that the provision of improved, dual-purpose chickens to vulnerable SFH is viable for economic growth, and resilience during emergencies in Nigeria.

Genetic and phenotypic relationships among feed efficiency, immune and production traits measured pre- (9–20 weeks of age) and post- (12 weeks from on-set of lay) maturity in indigenous chicken of Kenya were assessed by Miyumo et al. The genetic correlations obtained suggest that improved feed efficiency would be associated with high growth rates, early maturing chicken, high egg mass and reduced feed intake. Contrastingly, improved general keyhole limpet hemocyanin (KLH-IgM) and specific Newcastle disease virus (NDV-IgG) immunity would result in lower growth rates and egg mass but associated with early sexual maturation and high feed intake. This is an indication that indigenous chicken improvement programs should account for the potential genetic consequences of selective breeding for feed efficiency and immune-competence on production traits.

The study by Yussif et al. highlighted the phenotypic diversity and potential use of indigenous chicken breeds for breed improvement strategies in Uganda. Most of the chickens were raised under the scavenging as naked necks, frizzles, and polydactyls, within the Ugandan local chicken population even though some were rare. Certain alleles associated with traits such as tufted crests and rose combs were consistent with Mendelian expectations, whereas others, such as frizzles and polydactyl, had lower frequencies, implying possible feeding system in a mixed-crop livestock production system. A significant percentage of women (41%) were responsible for managing the chickens even though there were fewer (21%) female-headed households in Uganda. This was not unexpected as women are the primary keepers of smallholder poultry (Yakubu et al., 2020). The average flock size was 20, with hens laying approximately 40 eggs per year. The feather patterns, skin colours, earlobe sizes and colours, comb types, and beak shapes of indigenous chickens were all unique. The study indicated the presence of unique traits such endangerment. This study highlights the need for a careful balance between adaptability, farmer-preference and conservation of animal genetic resources in the sustainable development of local chickens in Uganda.

Performance of four distinct lines (L1, L2, L3, and L4) of Japanese quail (*Cortunix japanoica*) kept in the tropical climate of Tamil Nadu, India was investigated by Arunrao et al. The parameters measured were weekly body weight, daily feed consumption and egg production and mortality, if any, were recorded during the laying period. The weight of the eggs was measured once a week; in addition, age at sexual maturity, hen-day egg production, hen-housed egg production, livability, and feed efficiency in terms of feed per dozen eggs were calculated. It was observed on the average that Lines 3 and 4 outperformed others. The researchers, therefore suggest the selection of L3 and L4 for body weight and egg production in order to boost Japanese quail production in the tropics.

The smallholder poultry production must be maintained as an alternative source of food security and income in communities disturbed by various types of pollution in the environment. Among the different pollutants, the hydrocarbon contamination from oil spills, natural gas flaring, and organic pollutants (Ansah et al., 2022) is more prevalent and poses some major health and welfare difficulties in certain area around the word and the broad disruption of homeostasis caused by this pollutant puts the genetic potential of the birds at risk. In this regard, Oleforuh-Okoleh et al. examined role of genes and antioxidants in enhancing poultry’s tolerance to hydrocarbon toxicity. According to epidemiological research, they highlighted that the aryl hydrocarbon receptor (AhR) and nuclear factor erythroid 2p45-related factor 2 (Nrf2) genes, which regulate disease defense mechanisms, may be the cause of tolerance to hydrocarbon exposure. Different species may have different mechanisms and degrees of tolerance to hydrocarbon fragments, which could lead to differences in gene expression when exposed within members of the same species. They further emphasized that for the development of intensive, commercial, and financially viable smallholder poultry production in hydrocarbon-polluted communities, evaluation of the genetic architecture and risk assessment of diverse chicken breeds exposed to pollutants are essential.

The black-bone chicken (BBC) is an indigenous village chicken breed with unique properties that have contributed to its utilization and conservation. The fibromelanosis (Fm) locus on chromosome 20, which causes melanin hyperpigmentation, is primarily responsible for the distinct flavour and texture of BBC meat. Shinde et al. investigated the genetic basis of this trait in a variety of BBC breeds, including the Indian Kadaknath. According to the long-read sequencing data generated by the study, all BBC breeds have a complex chromosomal rearrangement at the Fm locus. The study clarified a previously debated scenario of chromosomal rearrangement and emphasised the significance of linkage in shaping genetic diversity. Compared to other BBC, the study revealed that Kadaknath had distinct genetic signatures in regions close to Fm, which could be related to immune function and disease resistance. The identification of specific genes with such genetic signatures for Kadaknath specific-phenotypes contributes to the genetic uniqueness of Kadaknath. The results provide insight into Kadaknath’s distinct genetics and immune modifications, thus suggesting a link between genetic diversity, artificial selection, and chromosomal rearrangements in domesticated species.

We are highly appreciative of the robust contributions of several authors to this Research Topic. It is our sincere hope that papers in this Research Topic would be found useful, while soliciting for more articles in the second volume which is currently open for submission in Frontiers in Genetics and Frontiers in Veterinary Science (Livestock Genomics Section).
